# Glycocalyx Degradation Induces a Proinflammatory Phenotype and Increased Leukocyte Adhesion in Cultured Endothelial Cells under Flow

**DOI:** 10.1371/journal.pone.0167576

**Published:** 2016-12-01

**Authors:** Karli K. McDonald, Scott Cooper, Lisa Danielzak, Richard L. Leask

**Affiliations:** 1 Department of Biomedical Engineering, McGill University, Montreal, Quebec, Canada; 2 Department of Chemical Engineering, McGill University, Montreal, Quebec, Canada; Ludwig-Maximilians-Universitat Munchen, GERMANY

## Abstract

Leukocyte adhesion to the endothelium is an early step in the pathogenesis of atherosclerosis. Effective adhesion requires the binding of leukocytes to their cognate receptors on the surface of endothelial cells. The glycocalyx covers the surface of endothelial cells and is important in the mechanotransduction of shear stress. This study aimed to identify the molecular mechanisms underlying the role of the glycocalyx in leukocyte adhesion under flow. We performed experiments using 3-D cell culture models, exposing human abdominal aortic endothelial cells to steady laminar shear stress (10 dynes/cm^2^ for 24 hours). We found that with the enzymatic degradation of the glycocalyx, endothelial cells developed a proinflammatory phenotype when exposed to uniform steady shear stress leading to an increase in leukocyte adhesion. Our results show an up-regulation of ICAM-1 with degradation compared to non-degraded controls (3-fold increase, p<0.05) and we attribute this effect to a de-regulation in NF-κB activity in response to flow. These results suggest that the glycocalyx is not solely a physical barrier to adhesion but rather plays an important role in governing the phenotype of endothelial cells, a key determinant in leukocyte adhesion. We provide evidence for how the destabilization of this structure may be an early and defining feature in the initiation of atherosclerosis.

## Introduction

Atherosclerosis is an inflammatory disease underlying the majority of cardiovascular related deaths [1]. Endothelial cell (EC) dysfunction and leukocyte adhesion are early markers in disease initiation. There are several key steps involved in leukocyte adhesion to the surface of the endothelium [2]. For example, inflammatory activation of the endothelium results in the up-regulation of several cell adhesion molecules and the secretion of a variety of chemokines, which promote leukocyte recruitment. Free-flowing leukocytes are captured from the blood stream and undergo rolling under flow conditions along the endothelium through the transient interactions between leukocyte glycoprotein (e.g. PSGL-1) and members of the selectin family P- and E-selectin [2,3] [4,5]. These interactions result in a reduction in the rolling velocity of leukocytes, enabling cells to receive signals deriving from chemokines presented on the luminal surface of cell [6]. Leukocyte microvilli flattening also serves to slow rolling and enhance the availability of chemokine receptors and integrins for interactions with their respective EC ligands [7]. Chemo-attractant activation of G-protein coupled receptors (GPCR) activates leukocyte integrins, which form high-affinity bonds with EC ligands such as intracellular adhesion molecule-1 and -2 (ICAM-1 and ICAM-2) and vascular cell-adhesion molecule (VCAM-1), resulting in slow rolling and firm adhesion. [8–10]. Arrested leukocytes can then crawl along the cell surface in search of exit cues where they exhibit transendothelial migration, the final step in the process [11].

Shear stress (SS) is the force acting on the surface of the cell and not only influences the physical contact between leukocytes and their counter-receptors, but is also key in regulating EC phenotype. Exposure of ECs to uniform, steady, laminar SS (LSS) leads to a more atheroprotective cell phenotype with a reduction in leukocyte adhesion and altered adhesion molecule expression [12–15]. Conversely, exposure to disturbed flow, characterized by SS gradients (both temporal and spatial), leads to a proinflammatory cell phenotype and atherosclerotic plaque development [16,17]. Therefore, understanding the interplay between hemodynamic forces, EC dysfunction and adhesion is key to furthering insights into the mechanism underlying the initiation and development of vascular pathologies such as atherosclerosis.

The luminal surface of ECs is covered by a carbohydrate-rich structure called the glycocalyx. Although largely understudied for many years due the difficulty in preserving this structure *in vitro*, it is becoming increasingly clear that the glycocalyx is key in regulating several EC functions. Specifically, the glycocalyx plays a role sensing and transducing mechanical forces into biochemical signals, and thus maintaining EC health. Several studies have shown that when the glycocalyx is disrupted, ECs can no longer adapt a healthy phenotype in response to flow [18–20]. Thi et al showed that the redistribution of F-actin fibers is blocked with glycocalyx disruption under flow [19]. The authors also demonstrate a role for the glycocalyx in gap junction reorganization and nitric oxide (NO) production [19]. Similarly, removal of the glycocalyx impairs the ability of ECs to sense flow and modulate migration speed and proliferation rate [21,22]. Due to its unique structure and location, the glycocalyx has also been proposed to play a role in adhesion [23]. To date, the central hypothesis is that it plays a structural role, impeding adhesion by covering adhesion molecules on the surface of the cell and by creating steric hindrance, making leukocyte binding more challenging. In the current study, we demonstrate that the glycocalyx plays more than a structural role in adhesion where degradation leads to an increase in leukocyte adhesion by inducing a proinflammatory phenotype marked by an increase in ICAM-1 expression and NF-κB activity under flow conditions. Such findings have direct relevance to atherosclerosis since a reduction in glycocalyx expression has been reported in areas of disturbed flow and therefore may provide a mechanism for how the demise of this structure could potentially lead to the predisposition of ECs to disease onset. Further, we provide a pathway for the signaling cascade we believe is responsible for this proinflammatory phenotype by linking a reduction in shear-induced eNOS expression to the unmitigated NF-κB activation and thus resultant EC inflammation.

## Materials and Methods

### Cell Culture

Human abdominal aortic endothelial cells (HAAECs; ATCC, CRL-2472, Coriell) were cultured in endothelial growth medium, supplemented with 10% fetal bovine serum and 1% penicillin streptomycin (Invitrogen) in tissue culture flasks coated with 0.1% pig gelatin at 37°C and 5% CO_2_. At confluence, cultures were rinsed with phosphate buffered saline solution (PBS) and harvested with 0.25% Trypsin-EDTA. Experiments were performed on cells at passage number 5.

### Enzymatic degradation

HAAECs were treated with 180 mU/ml of F. *heparinum* heparinase III (Sigma) for 2 hours. Heparinase III was diluted in serum free media. Degradation was quantified from immunofluorescent staining of heparan sulfate (HS) and Zeiss 510 laser scanning confocal microscopy. Analysis of acquired images was performed using a protocol previously published [24].

### Shear Application

Detailed protocols for the 3D cell culture models and flow apparatus have been described previously [16,25–27]. Briefly, straight tube models (hemodynamic parameters within the model are summarized in Table 1) were made by mounting polished stainless steel rods into pre-designed molds. Silicon elastomer (Sylgard 187, Dow Corning) was then added and cured around the rods. After polymerization, the rods were removed and the models were then prepared for cell culture by sterilizing and coating with fibronectin (Sigma F0895, 40μg/ml) in PBS and rotated overnight at 37°C. The fibronectin was then rinsed and HAAECs were seeded at a density of 1.25x10^6^ cells/ml. HAAECs were incubated on the rotator for 48 hours to achieve even coverage and confluence. Cells were then either exposed to up to 24 hours of 10 dyne/cm^2^ unidirectional LSS or statically cultured.

**Table 1:** Hemodynamic parameters in 3-D straight tube models.

Parameter	Straight tube model
Viscosity (cP)	0.975
Density (kg/cm^3^)	.9982
Wall shear stress (dyne/cm^2^)	10
Channel diameter (cm)	0.2
Flow rate (mL/min)	48.2
Reynolds number (Re)	525

### Protein collection and western blot

HAAECs were harvested from the models using a 0.25% trypsin-EDTA solution and spun at 1200 RPM for 5 minutes. Cells were then rinsed in cold PBS, spun at 3000 RPM for 5 minutes and lysed in cold RIPA Lysis Buffer (50mM Tris-HCl pH 6.8,150mM NaCl, 1% NP-40, 0.5% Sodium Deoxycholate, 0.1% SDS) with 0.1% protease inhibitor cocktail. A final spin at 13000 RPM was performed for 10 minutes. Western blots were performed using the Novex Protein Kit (Life Technologies). 10–15μg of protein was loaded on pre-cast Bolt 4–12% Bis-Tris Plus 10 well gels. Following the transfer, membranes were blocked with 5% nonfat dry milk in 0.1% Tween-20 solution in PBS (PBS-T) for 30 minutes. Primary antibodies were diluted in blocking buffer and incubated at 4°C overnight on a shaking plate. Antibodies consisted of ICAM-1 (1:100, Santa Cruz, sc-8439, monolconal), GAPDH (1:5000, Santa Cruz, sc-32233, monolconal), eNOS (1:100, Santa Cruz, sc-654, polyclonal) and IκB-α (1:100, Abcam, 32518, monoclonal). After three washes in 0.1% PBS-T, horseradish peroxidase (HRP) secondary antibodies (Jackson ImmunoResearch) diluted in blocking buffer were added at 1:5000 for 1 hour at room temperature (RT). The membranes were washed and detection was accomplished with the enhanced chemiluminescence method (Thermo Scientific) and UVP Biospectrum^®^ 810 MultiSpectral Imaging System. Densitometry for protein quantification was performed in ImageJ and data was normalized to the loading control.

### Adhesion Assays

Acute promyelocytic leukemia (NB4) cells were cultured in suspension in T75-flasks at 2x10^5^-1x10^6^ cells/mL in RPMI 1640 medium with 2 mM L-glutamine supplemented with 10% FBS and 1% penicillin streptomycin. For differentiation into granulocytes, cells were stimulated for 48 hours in the presence of 10^−6^ M all-*trans*-retinoic acid (ATRA; Sigma). Prior to adhesion assays, HAAECs were stimulated with tumor necrosis factor-alpha (TNF-α) for 24 hours at 10 ng/mL (Chemicon). NB4 cells were allowed to adhere statically at a concentration of 5x10^5^ on HAAECs or perfused at an inlet SS of 1.25 dyne/cm^2^ for 1 hour. Non-adherent cells were removed by washing the models with growth medium three times. The number of adherent NB4 cells was determined by manually counting cells from light microscope images at a magnification of 10x. For adhesion assays with ICAM-1 blocking, HAAECs were treated with 20 μM of mouse anti-ICAM-1 antibody (Santa Cruz, sc-8439, monoclonal) for 1 hour prior to the addition of NB4 cells. For adhesion assays with L-NAME, HAAECs were treated with 5 μM of N-Nitro-L-Arginine methyl ester hydrochloride (L-NAME, Sigma, N5751) for 48 hours prior to the addition of NB4 cells or protein collection.

### Morphology

The models were fixed in 1% paraformaldehyde (PFA)/PBS and rinsed three times in PBS. Cell morphology was assessed by adding 4% crystal violet (BD Biosciences) to the cells. After 5 minutes of staining, the cells were rinsed and imaged using light microscopy at 100x magnification (Leica DMIL microscope and Leica DC300 camera). Morphology was quantified using a previously developed protocol using Matlab^TM^ software [26]. Briefly, the shape index (SI) is a previously defined parameter used to characterize the degree of cell elongation where the SI of a circle is equal to 1 and a straight line is equal to 0.

### Immunofluorescence staining and confocal microscopy

HAAECs were fixed *in situ* in 1% PFA/PBS and blocked with 2% normal donkey serum (NDS) in PBS. For surface staining, primary antibodies (HS, 1:100, Millipore MAB2040 and ICAM-1, 1:200, Santa Cruz sc-8349 monoclonal) were diluted in blocking buffer and incubated overnight at 4°C on a shaking plate. Models were then rinsed three times in PBS and then incubated with the secondary antibody (1:600, Alexa Fluor 488 Anti-Mouse IgG, Molecular Probes, A21206) for 1 hour at RT. For NF-κB, cells were permeabilized with 0.2% Triton X-100 (Sigma), followed by blocking in 2% NDS and incubated overnight at 4°C with the NF-κB (p65) primary antibody (1:100, Invitrogen, 339900) diluted in 1% NDS/0.05% Triton X-100/PBS. The models were then incubated with the secondary antibody (1:600, Alexa Fluor 488, Molecular Probes, A21206) for 1 hour at RT. Nuclear staining was performed using 2 mg/mL RNase (Sigma) for 30 minutes at 37°C, followed by the addition of TO-PRO-3 nuclear counterstain (1:1000, Invitrogen) for 20 minutes at RT. Models were cut and mounted using 0.2% Dabco/Glycerol (1:5, Sigma) and imaged via laser scanning confocal microscopy (Zeiss Exciter 510). Images were acquired at 10x magnification. NF-κB localization was determined based on the overlapping fluorescent signals between the p65 labeled subunit (*green*) and the nuclear stain (*red*). Models were fixed at different time points (t = 0, 1, 3, 6, 12, 24 hours) following the application of flow. Quantification of overlapping signals was performed using the Zen2008 software. A threshold for overlap between red and green channels was established using the histogram tool. The number of cells displaying a positive overlap was counted manually. A minimum of 4 images (with a minimum of 25 cells counted per image) per condition for at least n = 3 experiments were analyzed.

### Statistical Analysis

Statistical analysis was performed by nonparametric tests using GraphPad Prism^TM^ software. Mean values were compared using t-tests and two-way analysis of variance (ANOVA) followed by Bonferroni post-test with a 95% confidence interval. P values less than 0.05 were considered significant. Bar graphs represent mean values ± SEM for a minimum of 3 independent experiments.

## Results

### 24 hours of LSS is not sufficient to restore HS levels to control levels following enzymatic degradation

**Fig 1:**
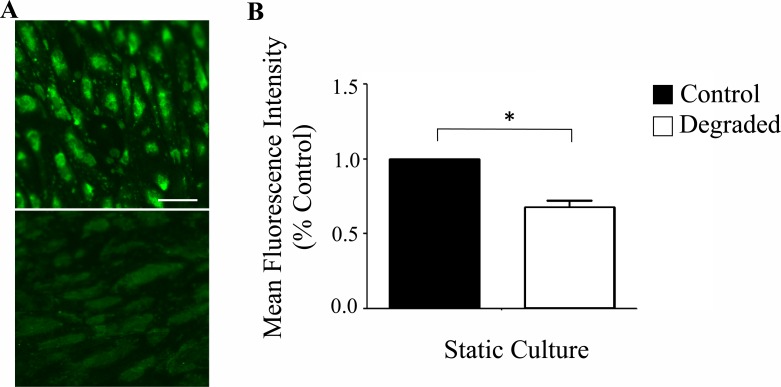
Glycocalyx establishment and degradation on HAAECs. (A) Representative confocal microscope images of HAAECs cultured statically and immunolabelled for HS. Images were acquired at 40x magnification (scale bar = 50μm). Top: Control; Bottom: Degraded. (B) Quantification of glycocalyx degradation from confocal images (n = 3, *p<0.05, error bars represent SEM).

**Fig 2:**
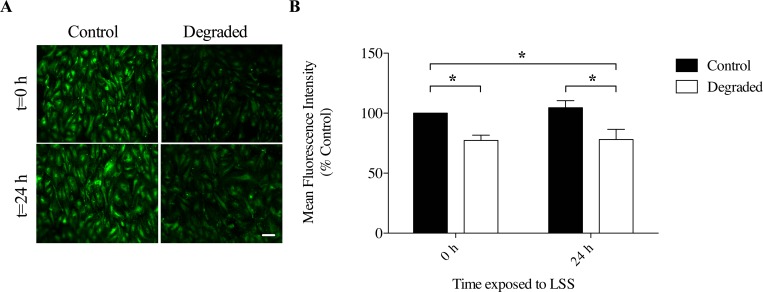
HS expression with degradation and 24 hours of LSS exposure in 3D cell culture models. (A) Representative confocal microscope images of HAAECs immunolabelled for HS and exposed to 10 dyne/cm^2^ LSS for 24 hours (t = 24h) or fixed directly after degradation (t = 0). Images were acquired at 10x magnification (scale bar = 50μm). (B) Quantification of HS expression after 24 hours of flow and degradation (n = 3, *p<0.05, error bars represent SEM).

We first established the presence of a glycocalyx on our HAAEC line through indirect immunofluorescence staining of one the main components (heparan sulfate; HS, Fig 1A and 1B). Next, we established conditions to enzymatically degrade the glycocalyx. With a 2hr incubation of cultured HAAECs with 180mU/mL *heparinase III*, we achieved ~30% reduction in HS expression under static conditions (Fig 1C). However since the majority of our experiments would be performed after 24 hours of LSS, we quantified the expression of HS following degradation with and without the application of 24 hours of LSS at 10 dynes/cm^2^ (Fig 2A). Our results show that HS expression is significantly reduced at both t = 0h and t = 24h compared to non-degraded control at t = 0h (22.3% ± 4.5 vs 22% ± 8.5, p<0.001, Fig 2B). Therefore 24 hours of LSS is not sufficient to restore HS levels to control levels following enzymatic degradation in our cell culture model and cell line.

### Glycocalyx degradation leads to an impaired morphological response in response to LSS

**Fig 3:**
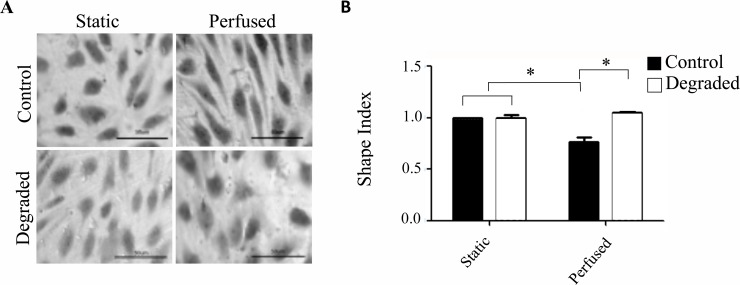
Morphological response of HAAECs with degradation and 24 hours of LSS in 3D cell culture models. (A) Representative light microscope images of HAAECs stained with crystal violet. Images were acquired at 40x magnification (scale bars = 50μm). (B) Quantification of cell morphology based on the shape index and normalized to static control (n = 3, *p<0.05, error bars represent SEM).

Elongation and alignment in the direction of flow is a well-characterized response of ECs exposed to LSS and the glycocalyx has been shown to play a key role in mediating this morphological adaptation [19]. In order to confirm that our degradation treatment was capable of eliciting a dysfunctional response consistent with other *in vitro* studies, we assessed the morphological response of HAAECs by quantifying the shape index with 24 hours of flow at an inlet SS of 10 dyne/cm^2^ compared to static controls, with and without *heparinase III* treatment. Treatment and flow conditions were normalized to the static control. Under static conditions, both control and HS-degraded HAAECs showed a cobblestone appearance with no specific orientation (Fig 3A). With flow, cells in the control condition became more elongated as expected with a mean shape index that decreased ~1.25 fold (p<0.05, Fig 3B). Cells treated with *heparinase III* however, did not elongate with flow and maintained a shape index comparable to static conditions. This finding is in agreement with previously published studies and validates our treatment condition.

### There is an increase in NB4 firm adhesion with glycocalyx degradation

**Fig 4:**
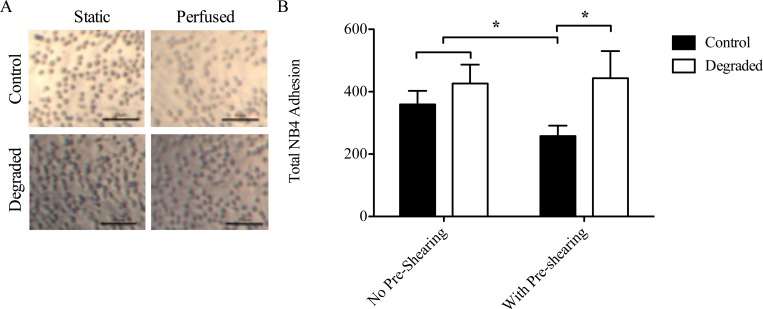
NB4 cell adhesion in 3-D cell culture models with degradation and flow. (A) Representative light microscope images of NB4 cells adhered to HAEECs in models. Images were acquired at 10x magnification (scale bars = 25μm). (B) Quantification of adherent NB4 cells (n = 3, *p<0.05, error bars represent SEM).

To determine the role of the glycocalyx in leukocyte adhesion, we performed adhesion assays on a TNF-α stimulated endothelium where ECs were exposed to either 24 hours of pre-shearing at an inlet SS of 10 dynes/cm^2^ or no pre-shearing (i.e. 24 hours of static culture, followed by the perfusion of leukocytes for 1 hour at an inlet SS of 1.25 dyne/cm^2^). These experiments were performed either with or without a degraded glycocalyx. The number of adherent leukocytes was quantified from light microscope images taken directly from the models after fixation (Fig 4A). Our results show that under control conditions, there was an ~1.5 fold reduction in leukocyte adhesion with pre-shearing compared to no pre-shearing (p<0.05, Fig 4B). These findings support the known atheroprotective effect of applied LSS on ECs. With degradation however, there was no reduction in adhesion and the number of adherent leukocytes remained similar to static control levels (Fig 4B). Considering flow conditions only, there was a 1.7-fold increase in leukocyte adhesion between control and degraded HAAECs. Therefore, glycocalyx disruption inhibits the shear-mediated reduction in adhesion observed under control conditions.

### There is no difference in NB4 adhesion with glycocalyx degradation under static conditions

**Fig 5:**
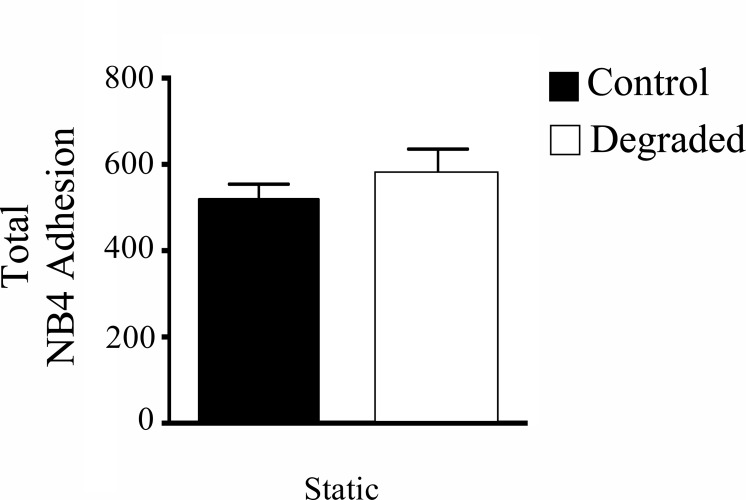
NB4 adhesion in static culture with degradation. HAAECs were cultured statically and NB4 cells were added statically following degradation (n = 3, p = 0.23, error bars represent SEM).

To verify whether the changes in adhesion with flow were from a structural disturbance of the glycocalyx (i.e. a decrease in steric hindrance), we performed adhesion assays under static conditions (no pre-shearing) with the addition of NB4 cells also performed statically. The number of adherent leukocytes on control versus degraded HAAECs was quantified from light microscope images. Interestingly, we found no significant difference in adhesion under static conditions between control and glycocalyx degradation (Fig 5). Therefore, altering the structure of the glycocalyx alone is not sufficient to induce NB4 adhesion to the endothelium.

### Glycocalyx degradation results in the up-regulation of ICAM-1 expression under flow

**Fig 6:**
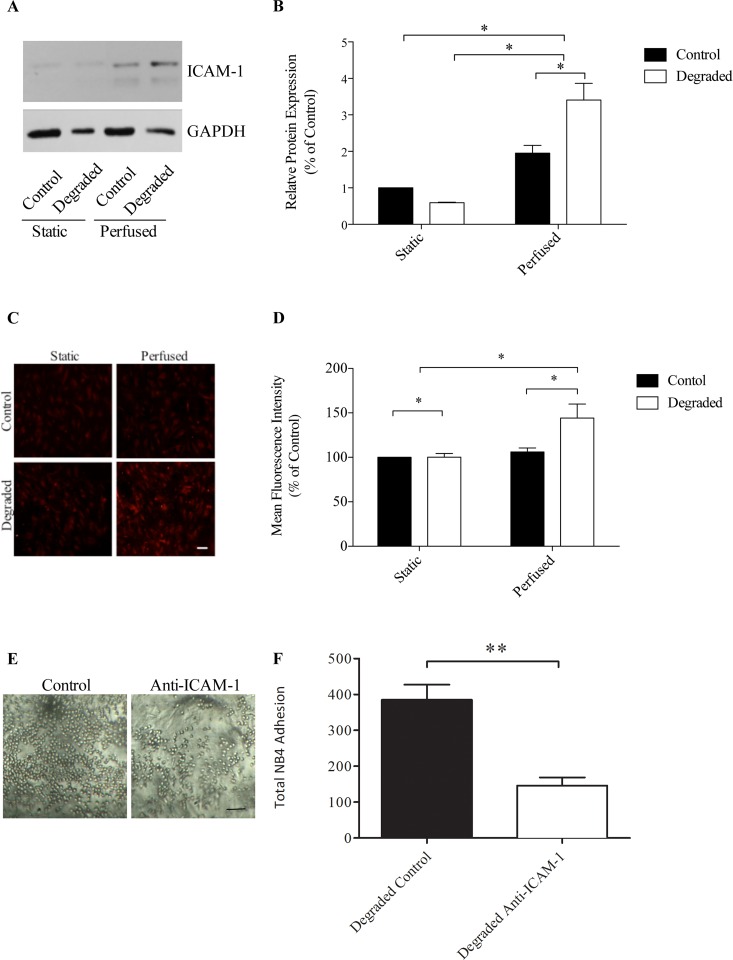
ICAM-1 protein expression with flow and degradation. (A) Representative western blot images showing ICAM-1 with GAPDH as the loading control. (B) Quantification of western blots using densitometry (n = 4, *p<0.05, error bars represent SEM). (C) Representative confocal microscope images of HAAECs immunolabelled for ICAM-1. Images were acquired at 10x magnification (scale bar = 50μm). (D) Quantification of ICAM-1 on the surface of HAAECs (n = 3, *p<0.05, error bars represent SEM). Perfusions were performed for 24 hours at an inlet SS of 10 dynes/cm^2^. (E) Representative light microscope images of NB4 cells adhered to HAEECs in models under control and ICAM-1 antibody blocking conditions under flow. Images were acquired at 10x magnification (scale bars = 25μm). (F) Quantification of adherent NB4 cells (n = 3, **p<0.01, error bars represent SEM).

To investigate the molecular mechanisms underlying the increase in adhesion with degradation under flow, we examined the expression of ICAM-1, an important molecule involved in the firm adhesion of leukocytes. Protein extracts were collected from both control and *heparinase III* treated cells with either static or perfused preconditioning and analyzed via western blot using an antibody specific to ICAM-1 (Fig 6A). Quantification was performed via densitometry and ICAM-1 bands were normalized to the loading control, GAPDH. Our results show that when HAAECs were exposed to 10 dynes/cm^2^ of LSS for 24 hours, there was ~50% up-regulation in ICAM-1 expression compared to static controls (p<0.05, Fig 6B), which is consistent with other published reports [28]. When we degraded the glycocalyx and applied flow, ICAM-1 expression was up-regulated by ~300% compared to the static control. Under flow conditions alone, there was a 1.8-fold increase in ICAM-1 between non-degraded and degraded HAAECs (p<0.05, Fig 6B). Of note, there was no significant difference in ICAM-1 expression with degradation under static conditions. We next verified whether there was any change in the expression of ICAM-1 on the surface of HAAECs. We performed IF staining for surface ICAM-1 and found a significant increase of ~30% in ICAM-1 with degradation and flow compared to control conditions (p<0.05, Fig 6C and 6D). We believe this spike in ICAM-1 protein levels and the concomitant increase in ICAM-1 on the surface may provide a possible mechanism for the increase in adhesion we observed previously. To test this hypothesis, we performed adhesion assays with degradation and flow on control and with ICAM-1 antibody blocking (Fig 6E and 6F). We found a significant reduction in the number of adherent NB4 cells when ICAM-1 was blocked compared to control (385±42 vs 146±23, p = 0.01). Similar results were found with degradation and ICAM-1 antibody blocking (144±28, data not shown).

### NF-κB response to LSS is de-regulated with glycocalyx degradation

**Fig 7:**
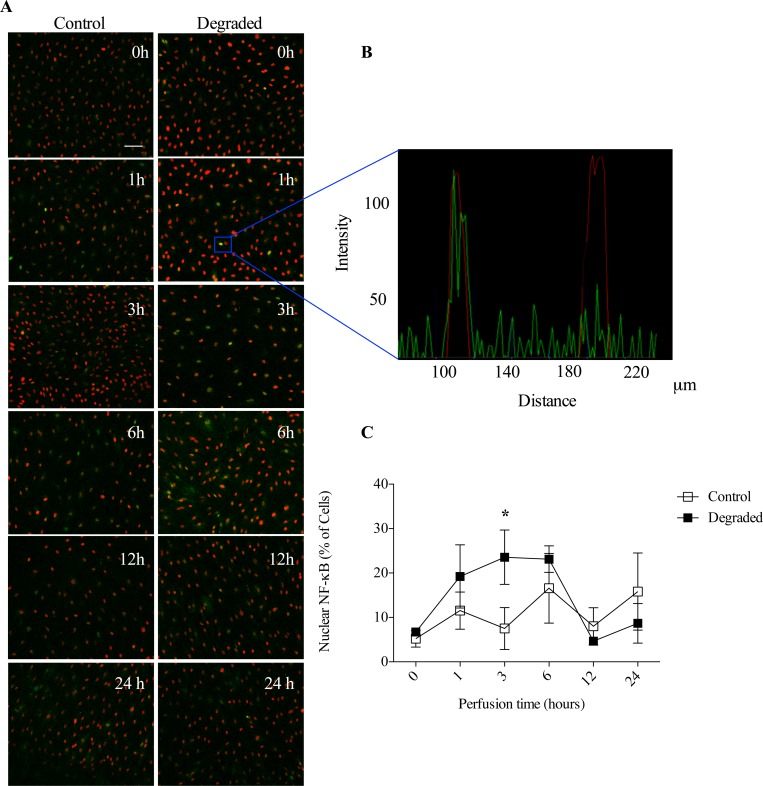
NF-κB response with flow and degradation. (A) Representative images from confocal microscopy of NF-κB (*green*) and nuclear counterstain (*red*) following exposure to different flow times. Images were acquired at 10x magnification (scale bar = 50μm). (B) Representative histogram showing a cell with nuclear NF-κB localization (overlap of *green* and *red* signals) versus a cell without NF-κB in the nucleus (no *green* overlap). These graphs were used to quantify the number of cells with NF-κB in the nucleus. (C) Quantification of the number of cells with nuclear NF-κB over time in either control or degraded conditions (n = 3, *p<0.05, error bars represent SEM).

To further elucidate the molecular mechanisms underlying our previous findings, we assessed NF-κB activity. In these experiments, we determined the number of cells with nuclear NF-κB by quantifying those which displayed overlapping fluorescence signals between the immunolabelled NF-κB p65 subunit (*green*) and a nuclear specific stain (*red*; Fig 7A). Images were collected via laser scanning confocal microscopy and analyzed using Zen^TM^ software (Fig 7B). We assessed nuclear translocation at different time points (t = 0, 1, 3, 6, 12, 24 hours) following the application of flow since studies have shown that NF-κB activation is transient with a recurrent nuclear/cytoplasmic pattern that occurs over time with LSS [29]. Our results are consistent with these observations in that there was an increase in the number of cells with nuclear NF-κB at t = 1h, a decrease at t = 3h, an increase at t = 6h, a decrease at t = 12h and another increase at t = 24h in response to 10 dynes/cm^2^ LSS (Fig 7C). When HAAECs were first treated with *heparinase III* however, we found that this response was impaired. Our data shows an increasing number of cells with nuclear NF-κB at t = 1h compared to control (19.3% ± 7.1 vs 11.3% ± 4.1), reaching a peak at t = 3h (23.2% ± 6.1 vs 7.5% ± 4.7), which is sustained at t = 6h (23.13% ± 2.9 vs 16.5% ± 7.8). The number of positive nuclear NF-κB cells returned to baseline levels at t = 12h (4.65% ± 0.2 vs 8.4% ±4.1) with only a small increase at t = 24h (8.7% ± 4.4 vs 15.8% ± 8.6). Therefore, not only is the normal cyclical response to flow deregulated with degradation, there is also a significant increase of ~3-fold in the number of cells with nuclear NF-κB at t = 3h (p<0.05). As a major marker and regulator of EC inflammation, such NF-κB results lend support to our hypothesis that the glycocalyx plays a role in mediating adhesion by modulating the EC proinflammatory phenotype in response to flow.

### eNOS up-regulation in response to LSS is inhibited with glycocalyx degradation

**Fig 8:**
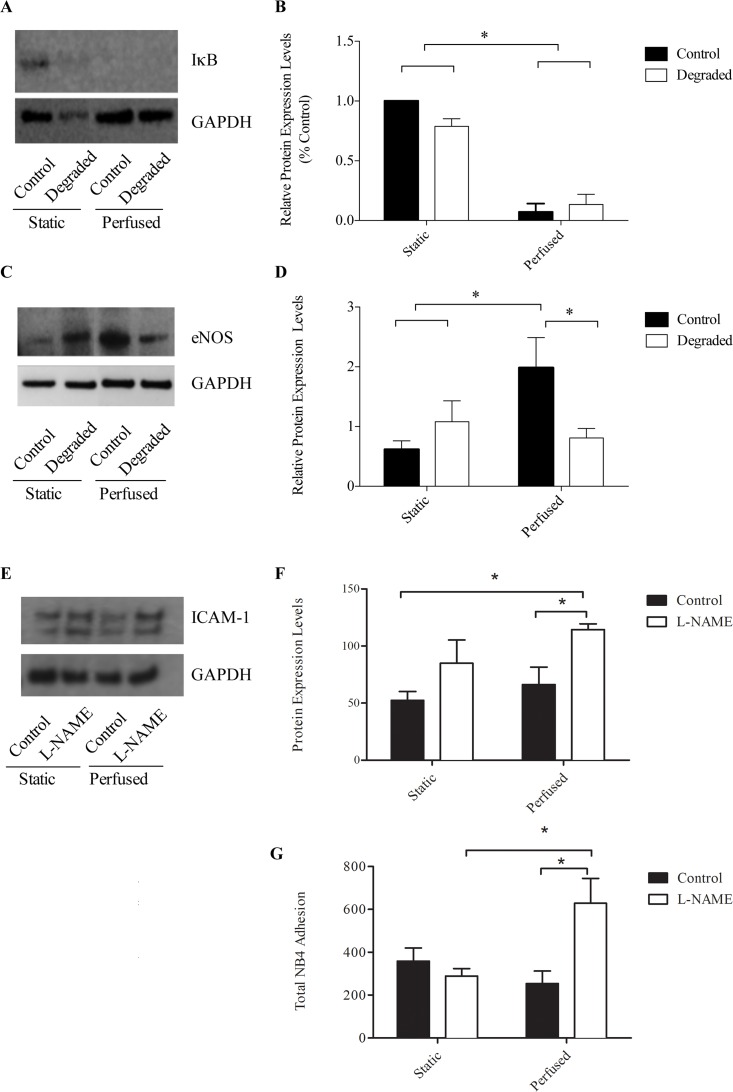
IκB and eNOS protein expression with flow and degradation. (A and C) Representative western blot images showing IκB and eNOS with GAPDH as the loading control. (B and D) Quantification of western blots using densitometry (n = 4, *p<0.05, error bars represent SEM). Perfusions were performed for 24 hours at 10 dynes/cm^2^. (E) Representative western blot images showing ICAM-1 with GAPDH as the loading control in the presence of the NO inhibitor, L-NAME. (F) Quantification of western blots using densitometry (n = 3, *p<0.05, error bars represent SEM). (G) Quantification of adherent NB4 cells with and without L-NAME and under flow conditions (n = 3, *p<0.05, error bars represent SEM).

Our previous findings indicated that it was not degradation of the glycocalyx itself that led to an altered cell phenotype, but rather the inability of cells to sense and transduce the LSS required to maintain a healthy phenotype. Therefore, we aimed to identify a shear-sensitive molecule that could be responsible for the down-stream effects observed. NF-κB activity is primarily regulated via two distinct mechanisms: one involving IκB and the other involving NO. Therefore, we examined the expression of both of IκB and eNOS via western blot analysis. Protein extracts were collected from both control and HS-degraded and static and perfused conditions and antibodies specific to either protein were used for immunoblotting with GAPDH as a loading control (Fig 8A). Quantification via densitometry revealed that upon exposure to 24 hours of 10 dynes/cm^2^ LSS, there was no significant difference in IκB protein expression levels between control and degraded conditions (Fig 8B). We observed a decrease in IkB expression with the application of flow regardless of treatment condition. Therefore, degradation does not appear to influence IκB expression. eNOS expression on the other hand showed a dramatic difference in expression with degradation upon flow exposure. With the application of 10 dynes/cm^2^ LSS, we observed an ~3-fold up-regulation in eNOS levels compared to static controls (p<0.05, Fig 8C and 8D). With degradation however, this response to LSS was significantly blunted and eNOS levels remained similar to static control levels. Under flow conditions alone, degradation results in a 2.4-fold decrease in eNOS expression compared to non-degraded control. These findings are consistent with other published *in vitro* studies reporting diminished NO levels in response to flow with degradation [18,20]. Therefore, the glycocalyx is required for the LSS-induced up-regulation in eNOS. Furthermore, inhibition of eNOS levels may be responsible for the de-regulated NF-κB activity under flow with degradation resulting in the increase in ICAM-1 and adhesion.

To determine whether a reduction in NO with degradation did indeed play a role in the observed findings, we examined ICAM-1 protein expression and performed adhesion assays with and without the NO inhibitor, L-NAME. We found a significant up-regulation in ICAM-1 with L-NAME treatment compared to control under flow (66.31±15.23 vs 112.4±7.05, p<0.05, Fig 8E and 8F). For adhesion assays, there was a significant increase in the number of adherent cells with L-NAME treatment compared to control under flow conditions (253±59 vs 628±116, p<0.05, Fig 8G).

### Model

**Fig 9:**
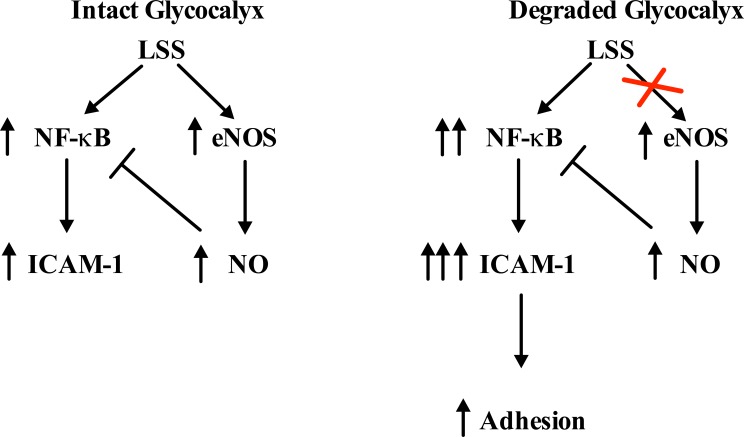
Model for the molecular adhesion pathway altered with degradation. Flow diagram illustrating how glycocalyx degradation interrupts the negative feedback loop regulating NF-κB activity. When the glycocalyx is degraded, NO levels are inhibited resulting in increased NF-κB activity. This results in the over-stimulation/activation of ECs evidenced by an increase in ICAM-1 and leukocyte adhesion.

A negative feedback loop has been proposed where upon LSS stimulation, NF-κB translocates to the nucleus, inducing the expression of multiple genes, including ICAM-1 and eNOS. Increased eNOS expression results in an increase in bioavailable NO production, which then inhibits NF-kB activation and DNA binding, allowing cells to return to an ambient state [30]. A reduction in NO production, such as with glycocalyx degradation, could therefore provide one pathway leading to the unmitigated activation of NF-kB, resulting in unopposed EC inflammation as indicated by increased ICAM-1 expression and leukocyte adhesion (Fig 9). This EC phenotype is characteristic of atheroprone regions of the vasculature [17]. Whether other factors involved in leukocyte adhesion are also altered with glycocalyx degradation warrants further investigation.

## Discussion

Leukocyte adhesion to an inflamed endothelium is a defining feature of atherosclerosis. The focal nature of this disease has been attributed to the dysfunction of ECs in vascular regions of disturbed flow, such as curvatures and bifurcations [31]. The glycocalyx has been shown to play an important role in both the sensing and mechanotransduction of SS forces and maintaining EC health. In the current study, we aimed to define the role of the glycocalyx in leukocyte adhesion to cultured ECs under uniform steady flow. We found that with the application of 24 hours of 10 dynes/cm^2^ uniform LSS there was a significant decrease in adhesion under control conditions (Fig 4A and 4B). However, when the glycocalyx was disrupted, HAAECs no longer adapted this atheroprotective phenotype (Figs 3, 6, 7 and 8) in response to flow and adhesion levels remained comparable to static control. Such results led us to investigate more closely the development of a proinflammatory cell phenotype with degradation under flow. Indeed, our results show that glycocalyx disruption leads to an up-regulation in ICAM-1 protein expression and cell surface expression (Fig 6), increased NF-κB activation (Fig 7A and 7B) and also a deregulation in the normal cyclical response of NF-κB to LSS (Fig 7B). We present a model describing how the induction of this proinflammatory state may be mediated up-stream by the inhibition of shear-induced production of NO with degradation (Figs 8 and 9). Taken together, this data demonstrates that the glycocalyx is an important structure in governing both the inflammatory state of the ECs and the induction of atheroprotective phenotypes by LSS. Such findings have direct relevance to vascular pathologies such as atherosclerosis in which EC health is a key determinant in disease initiation and progression.

We performed our experiments in 3-D cell culture models where ECs were exposed to more realistic hemodynamic forces than traditional parallel plate experiments [25]. Previous studies by our group have demonstrated the effectiveness in using these models to expose ECs to LSS [16,25–27,32–36]. Specifically, HAAECs were exposed to 10 dynes/cm^2^ of LSS for 24 hours. Studies have shown that the structure of the glycocalyx is sensitive to flow where there is a positive correlation between glycocalyx thickness and the level of LSS [37]. Recently, Giantsos et al showed that 24 hours of 15 dynes/cm^2^ LSS can effectively restore glycocalyx expression to baseline levels following degradation [38]. The authors also show that the morphological response to flow, which is normally impaired with degradation, is also re-established upon exposure to 24 hours of LSS. Therefore, in the present study, we characterized glycocalyx expression with 24 hours of flow following enzymatic degradation in our models through the immunofluorescence staining of HS. Our results demonstrate that with an initial decrease in HS expression of ~27% (t = 0h), expression levels were not restored after 24 hours of applied LSS and remained reduced by ~28% (Fig 2B). We confirmed these results by showing that cells do not regain their ability to elongate with flow and remain in a rounded cobblestone appearance after 24 hours of flow with degradation (Fig 3A and 3B). These findings are in agreement with both *in vivo* and *in vitro* studies, which suggest that 5–7 days are required for the glycocalyx to make a full recovery following disruption [39,40]. Reported differences between the present study and that of Giantsos et al are likely the result of different degradation treatment conditions. Moreover, Giantsos et al did not report the initial level of degradation and therefore it is possible that we achieved a greater initial removal of HS from the surface of HAAECs. Indeed, our study employs a greater concentration and time of *heparinase III* treatment, and therefore may be inducing a more severe trauma from which it takes longer for cells to recover. We also employ models with a geometry that more closely resembles *in vivo* conditions (i.e. cells are cultured in a tubular 3D environment, preserving continuous cell-cell contact and eliminating boundary effects), which may account for some of the differences in our results.

The glycocalyx is an important structure in several EC responses to flow. An inability of ECs to respond to their external environment leads to EC dysfunction and vascular deterioration. Consequently, EC health is largely dependent on the proper sensing and transducing of hemodynamic forces. Recent evidence suggests that ECs exposed to uniform steady LSS, have activated intracellular signaling that leads to gene expression that is atheroprotective [41]. In contrast, EC dysfunction and subsequent atherosclerotic plaque formation is localized in areas of disturbed flow such as bifurcations, curves and branches [42]. For example, in the carotid artery, early atherosclerotic lesions are more frequently observed at the carotid sinus bifurcation, where the endothelium is exposed to disturbed flow [42]. Interestingly, these areas have also been shown to have reduced expression of the glycocalyx [43]. Moreover, Koo et al showed on cultured ECs that steady uniform LSS promotes the formation of a full and robust glycocalyx while exposure to non-laminar shear waveforms impedes this structural adaptation [40]. Therefore, the integrity of the glycocalyx may be an important factor when considering the mechanisms underlying the focal nature of disease development. In support of this, several studies have linked glycocalyx disruption with increased leukocyte adhesion, an early step in atherogenesis. Constantinescu et al showed that degradation of the glycocalyx *in vivo* stimulates the firm adhesion of leukocytes in the absence of changes in leukocyte rolling behavior [44]. Potter et al and Lipowsky et al showed that with either enzymatic degradation or via cytokine-induced shedding, there is an increase in leukocyte adhesion [23,39]. The results from our adhesion assays are in agreement with these *in vivo* studies as there is a significant increase in leukocyte adhesion with degradation and flow (Fig 4A and 4B). Our results show however, that by solely removing the glycocalyx, there was no increase in adhesion under static conditions (Fig 5). Therefore, describing the glycocalyx as merely a physical barrier to adhesion may not be accurate. We therefore hypothesized that the glycocalyx also mediates intracellular signaling pathways that affect adhesion levels. Indeed, we provide evidence for the first time that glycocalyx degradation actually elicits a proinflammatory EC phenotype through the up-regulation of ICAM-1, decreased eNOS expression and disrupted NF-κB signal transduction, which provides further insight into its role in adhesion.

ICAM-1 is a key molecule involved in the firm adhesion of leukocytes to the endothelium. ICAM-1 expression has been linked to inflammation and disease where strong expression of ICAM-1 has been detected in atherosclerotic lesions [45]. Moreover, this adhesion molecule is SS sensitive where physiological levels of LSS result in an increase in its expression compared to static controls [28]. In support of these findings, a putative SS responsive element has been identified within the ICAM-1 promoter, which is specifically activated by LSS [13]. Although ICAM-1 expression is increased with atheroprotective levels of LSS, it is likely that the other SS-induced atheroprotective signaling pathways are activated (i.e. the down-regulation of other proinflammatory genes and up-regulation of anti-inflammatory genes) which may abrogate the increased ICAM-1. Further, it is also possible that although ICAM-1 is up-regulated, levels may not reach a minimum threshold required to induce significant leukocyte binding. Lastly, while increased ICAM-1 levels have been reported for both mRNA and protein, it is also possible that this increased amount is not expressed at the surface of the cell where it can participate in adhesion. In the present study, we found that the application of LSS indeed results in the up-regulation of ICAM-1 by ~50% compared to static controls in our models (Fig 6B). With degradation however, we observed an up-regulation ~300% compared to controls under flow. Interestingly, this result was flow specific where under static conditions there was no significant difference in ICAM-1 expression between degraded and non-degraded conditions. Therefore, our results suggest that with degradation there is a disruption in the cell-signaling pathway mediating ICAM-1 expression. These results support the hypothesis that the glycocalyx is key in eliciting an atheroprotective response to flow and thus modulates endothelial inflammatory phenotype. Moreover, we show that ICAM-1 expression is also up-regulated on the surface of ECs with flow and degradation. The increase in ICAM-1 enhances the binding of leukocytes to ECs as demonstrated through a significant reduction in leukocyte adhesion when ICAM-1 is blocked (Fig 6E and 6F).

Activation of EC inflammation and expression of ICAM-1 are regulated via the transcription factor, NF-κB, which is also shear sensitive. In the inactive state, NF-κB subunits p50 and p65 are bound to the inhibitory protein IκB within the cytoplasm. Upon activation by a variety of stimuli, the IκB subunit is phosphorylated and degraded, allowing the p50/p65 dimer to translocate to the nucleus to transactivate a variety of genes [46]. Upon exposure to LSS, NF-κB cycles between the cytoplasm and nucleus [47]. Studies have shown that there is an increase in nuclear NF-κB immunofluorescence staining after 30 minutes of flow, reaching a peak at 1 hour, which then returns to the cytoplasm after 3 hours followed by nuclear localization after 6 hours [29,47]. These immunofluorescence experiments correspond to NF-κB DNA binding activity assays, which show a similar cyclical pattern [47]. This transient response is believed to be a protective mechanism ensuring cells are not over stimulated and can adapt and return to a quiescent state with changing conditions. By performing immunofluorescence time point experiments and quantifying nuclear NF-κB, we confirmed and extended these findings, by showing that the cyclical pattern of NF-κB continues for up to 24 hours with applied LSS (Fig 7B). NF-κB activity following degradation has not been previously reported. We show for the first time that when the glycocalyx is removed, there is a striking difference in the NF-κB response to flow. Our results demonstrate that there are more cells with nuclear NF-κB at 1 hour, which continues to rise over 3 hours and is sustained for up to 6 hours, until finally returning to control levels at 12 hours. Therefore, not only is there a burst of NF-κB in the nucleus early on, degradation also impairs the cyclical response at the earlier time points, where NF-κB remains in the nucleus. We believe this may result in cells becoming over stimulated and may lead to the spike in ICAM-1 we observed after 24 hours. Indeed, previous studies by our group have shown that it takes over 12 hours for ICAM-1 to be expressed following NF-κB induction [16]. Therefore, when the glycocalyx is removed, cells can no longer sense and respond normally to the ‘atheroprotective’ effects of applied LSS and an inflammatory response ensues. In order to identify this disconnect between the sensing of shear and the subsequent inflammatory response, we assessed eNOS expression.

NO production through increased eNOS gene expression is one of the fastest and earliest EC responses to flow [48]. Recently, it has been shown that the glycocalyx mediates shear-induced eNOS activation through its core protein GPC1, which is bound to caveolae where eNOS resides [49]. NO is well known for its role in regulating vascular tone, however this molecule also modulates inflammation and atherogenic endothelial activation through the inhibition of NF-κB activity [50]. Indeed, NO reduces NF-κB DNA binding through two distinct mechanisms: one through S-nitrosylation of Cys62 on the p50 subunit, preventing its nuclear translocation and the second through reduced degradation of the IκB inhibitory subunit [51]. NF-κB also binds the eNOS promoter, inducing the expression of multiple NOS isoforms, including eNOS and iNOS [52]. A negative feedback loop exists in which increased NO production, either via LSS induction or through the binding of NF-κB to the eNOS gene, then serves to inhibit NF-κB activity. We sought to determine eNOS expression with flow and degradation as a potential mechanism for the de-regulated NF-κB response we observed. Western blot analysis revealed a significant up-regulation in eNOS expression when cells were exposed to 24 hours of 10 dyne/cm^2^ LSS (Fig 7B and 7D). With degradation however, this response was blunted and eNOS levels remained comparable to static controls. This is in agreement with Florian et al who showed that NO production is abolished with glycocalyx disruption on cultured ECs after 30 minutes of 15 dyne/cm^2^ LSS [18]. Recently, it has also been shown that NO levels are reduced in rat mesentery microvessels with degradation [53]. Therefore, we believe there may be a reduction in the ambient levels of shear induced NO through the inhibition in eNOS expression with degradation, interrupting the negative feedback loop modulating NF-κB activity. Indeed the selective inhibition of NO using L-NAME resulted in an increase in ICAM-1 protein expression and leukocyte adhesion under flow compared to controls (Fig 8E–8G). These results were similar to those observed with glycocalyx degradation.

Many assumptions were made in this study to make it feasible. We have used *in vitro* dynamic cell culture models to model an *in vivo* response. We have used steady flow to condition with uniform laminar shear stress to condition our endothelial cells. We have also used a “neutrophil like” cell line, NB4s to model leucocyte attachment. ATRA-induced NB4 cells transition from the promyelocyte to the neutrophilic myelocyte stage. These cells have been shown to have a number of markers of the granulocytic lineage and increased expression of α4 (VLA4) and β2 (LFA-1, Mac-1) [54,55]. We have previously shown the utility of these cells in identifying regional variations in leukocyte attachment [34]. The glycocalyx in vivo is considerably thicker than that measured in vitro [56]. In addition, Zeng et al. have shown that S1P is necessary to ensure the health of the glycocalyx in vitro [57]. In our study, we used 10% FBS in our media concentration which is estimated to have sufficient S1P to ensure glycocalyx health [57]. Regardless of these limitations, when comparing our control and degraded glycocalyx results, we see a significant effect of degradation of the glycocalyx components on cell phenotype and leukocyte attachment. As with any *in vitro* cell culture experiment, the pathophysiological relevance of the findings can only be established when carefully analyzed in the context of other experimental and clinical data.

In conclusion the current study describes a novel role for the glycocalyx in leukocyte adhesion. We provide evidence for a pathway between shear sensing, the development of a proinflammatory phenotype and the functional consequence of increased adhesion to the endothelium with glycocalyx disruption. More specifically, we suggest that glycocalyx degradation, which occurs in atheroprone regions, interrupts the normal NO/NF-κB feedback loop, predisposing ECs to endothelial inflammation and atherosclerosis. We believe this delicate surface structure may be key in tipping the balance from an anti- to a proinflammatory EC phenotype. In the context of atherosclerosis, one can envision a scenario where over time, exposure of ECs to disturbed flow patterns eventually causes the gradual demise of the glycocalyx. Upon reaching a critical threshold, ECs can no longer respond and adapt to external hemodynamic forces properly. This results in decreased NO levels which then trigger an inflammatory response, ultimately leading to leukocyte adhesion and focal vascular inflammation. Strategies aimed at maintaining EC health and inhibiting adhesion, potentially through preserving the integrity of the glycocalyx, can perhaps mitigate some of the downstream pathways that eventually lead to disease initiation and development.
